# Small‐Bowel Capsule Endoscopy in Immune‐Mediated Enterocolitis: A Case Series of Four Patients

**DOI:** 10.1002/jgh3.70201

**Published:** 2025-06-18

**Authors:** Masayuki Orikasa, Tomoyoshi Shibuya, Hirotaka Ishino, Masashi Omori, Rina Odakura, Masao Koma, Kentaro Ito, Takafumi Maruyama, Kei Nomura, Dai Ishikawa, Mariko Hojo, Akihito Nagahara

**Affiliations:** ^1^ Department of Gastroenterology Juntendo University School of Medicine Tokyo Japan; ^2^ Department of Pathophysiological Research and Therapeutics for Gastrointestinal Disease Juntendo University School of Medicine Tokyo Japan; ^3^ Innovative Microbiome Therapy Research Center Juntendo University Tokyo Japan

**Keywords:** enterocolitis, immune‐mediated adverse events, immune‐related adverse events, small bowel endoscopy findings

## Abstract

Immune checkpoint inhibitors (ICIs) have emerged as a cornerstone of cancer immunotherapy but are associated with immune‐mediated adverse events (imAEs), including gastrointestinal toxicities. Among these, enteritis is a less common but clinically significant complication. However, small intestinal involvement remains under‐recognized, and endoscopic findings are not well characterized. We report four cases of imAE enteritis in which small‐bowel capsule endoscopy (SBCE) was performed. SBCE revealed variable mucosal abnormalities, including villous atrophy, aphthous ulcers, and extensive erosions; in one case, deep circumferential ulcers with stricture were observed. While one patient responded to corticosteroids, the other three required escalation to biologic therapy due to steroid‐refractory disease. These findings suggest that SBCE may play a valuable role in assessing disease extent and predicting treatment responsiveness in imAE enteritis. Early utilization of SBCE could facilitate timely therapeutic decision‐making in affected patients.

## 1 Introduction

ICIs exert their antitumor effects by blocking tumor cell‐mediated suppression of immune cells, and are effective in treating tumors that are resistant to conventional chemotherapy. However, various autoimmune complications, known as immune‐mediated adverse events (imAEs), can occur; among these, imAE enteritis is the second most frequent complication after cutaneous disorders. There are still few reports on the small‐bowel capsule endoscopy (SBCE) findings in patients with imAE enteritis. In this study, we present four patients with imAE enteritis who underwent SBCE, focusing on endoscopic findings.

### 1.1 Case 1

An 82‐year‐old man was diagnosed with bladder cancer with postoperative recurrence, multiple lung metastases, and lymph node involvement. Pembrolizumab therapy was initiated, and after 4 months, he developed approximately 10 episodes of watery diarrhea per day, accompanied by elevated inflammatory markers. The patient was admitted for further evaluation. Blood tests revealed mild inflammation, renal dysfunction, and hypokalemia. Stool examinations were negative for pathogenic bacteria, and Clostridioides difficile toxin was not detected. After admission, SBCE was performed, revealing whitish, villous‐like changes in a segment of the proximal small intestine; however, no other significant abnormalities were observed. Oral prednisolone at 25 mg was initiated for Grade 2 imAE colitis. The diarrhea gradually improved, and prednisolone was tapered accordingly. No recurrence of symptoms was observed, and the patient was discharged after completing a two‐month course of corticosteroid therapy.

### 1.2 Case 2

A 55‐year‐old woman was diagnosed with hepatocellular carcinoma and initiated on combination immunotherapy with durvalumab and tremelimumab. Ten days after the first administration, she developed fever and nausea and was transported to the emergency department. Blood tests revealed mildly elevated inflammatory markers and biliary enzymes. Stool culture and *C. difficile* toxin tests were negative. After hospitalization, colonoscopy and SBCE were performed. Colonoscopy revealed multiple erosions and mucosal erythema throughout the colon, while SBCE showed erosions and coarse mucosa extending from the duodenum to the jejunum. Intravenous prednisolone 50 mg was initiated for Grade 3 imAE colitis. However, symptoms did not improve with steroid tapering, and the condition was diagnosed as steroid‐refractory colitis. Infliximab (IFX) was administered, resulting in rapid resolution of diarrhea and abdominal pain. Only one dose of IFX was given, and prednisolone was tapered off successfully thereafter.

### 1.3 Case 3

A 67‐year‐old man with hepatocellular carcinoma received 10 cycles of atezolizumab and bevacizumab, after which his regimen was switched to durvalumab and tremelimumab. Ten days after the switch, he developed 15–20 episodes of watery diarrhea per day, prompting hospitalization. Blood tests revealed a mild inflammatory response and elevated liver transaminases. Stool cultures and *C. difficile* toxin tests were negative. Esophagogastroduodenoscopy (EGD), colonoscopy, and SBCE were performed. EGD showed erythema and multiple erosions on the duodenal mucosa. Colonoscopy revealed diffuse mucosal irregularity with purulent exudates resembling ulcerative colitis throughout the entire colon. SBCE revealed multiple aphthous ulcers with surrounding erythema throughout the small intestine. Duodenal biopsy showed lymphocytic infiltration and crypt epithelial apoptosis. Immunohistochemistry demonstrated infiltration of CD3+, CD4+, and CD8+ T cells in the epithelium. Intravenous prednisolone 40 mg was started for Grade 3 imAE colitis. Although there was a temporary reduction in diarrhea, a flare occurred during steroid tapering. Steroid‐refractory colitis was diagnosed, and IFX was initiated, resulting in a rapid improvement. IFX was administered at weeks 2 and 6, and the treatment course was completed without further relapse during prednisolone tapering.

### 1.4 Case 4

A 60‐year‐old man was diagnosed with liver metastases and carcinomatous pleuritis secondary to squamous cell lung carcinoma. He was initially treated with carboplatin, paclitaxel, and pembrolizumab. After 7 cycles, ICIs were discontinued and the regimen was switched. Six months later, the patient developed fever and frequent diarrhea, leading to hospitalization. Blood tests revealed markedly elevated C‐reactive protein (CRP), with no other significant abnormalities. Stool cultures and *C. difficile* toxin tests were negative. Colonoscopy revealed diffuse loss of vascular pattern and coarse mucosa throughout the colon. Although no overt ulcerations were seen, some areas showed white exudate. Intravenous prednisolone 50 mg was initiated, but symptoms persisted, prompting IFX administration. However, after two doses of IFX, only minimal improvement was observed. SBCE was subsequently performed, revealing multiple erosions and erythema throughout the small intestine, along with circumferential ulcers and villous atrophy. A deep ulcer with circumferential stricture was observed at the terminal ileum, although capsule passage was not obstructed. Due to inadequate response to IFX, vedolizumab (VDZ) was initiated. Following VDZ administration, gradual improvement in diarrhea was noted. A second dose was administered 2 weeks later, and a follow‐up SBCE showed overall mucosal healing and improvement of the terminal ileum ulcer. A third VDZ dose was given, after which the patient's diarrhea resolved completely.

## 2 Discussion

**FIGURE 1:**
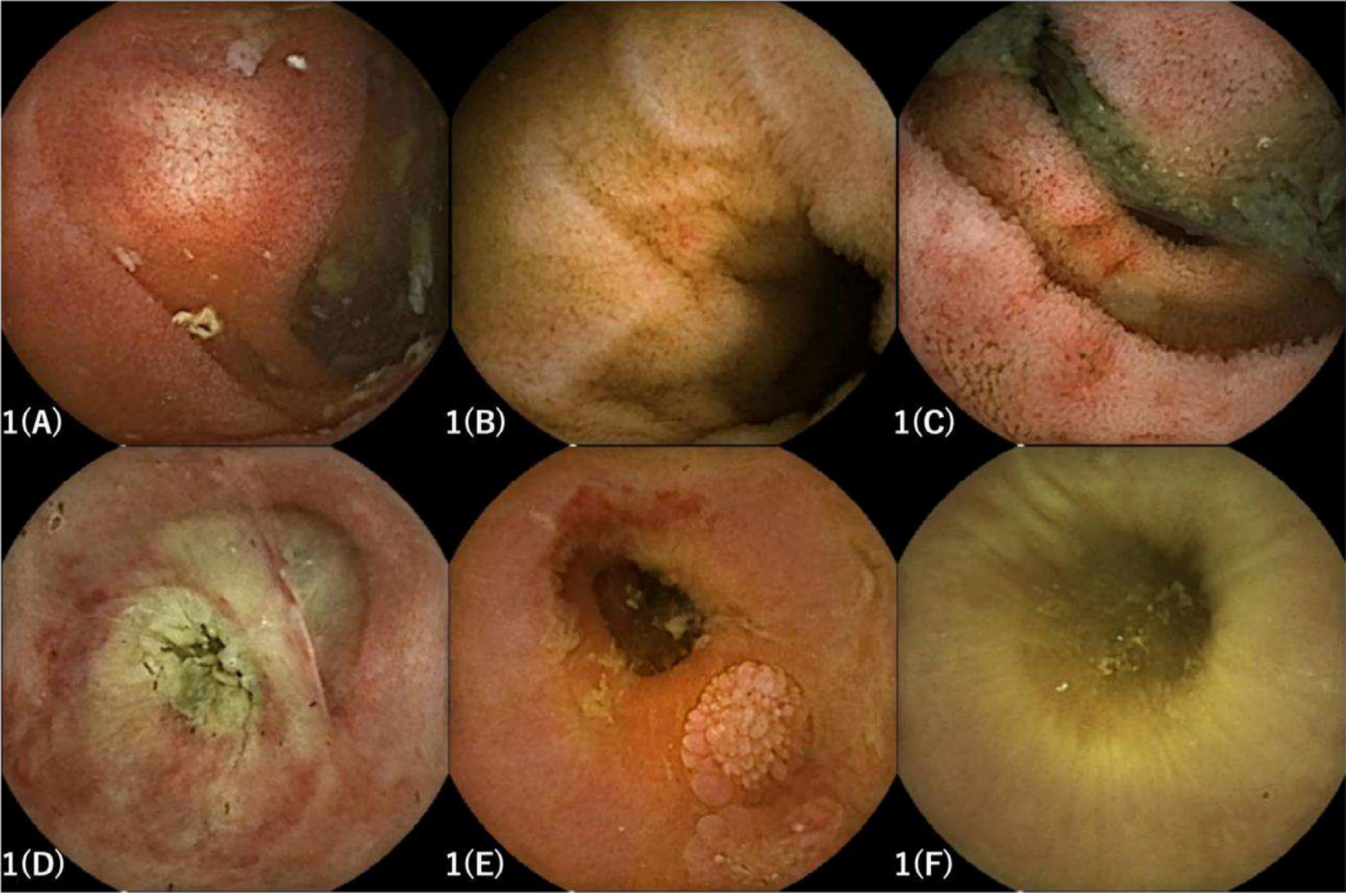
SBCE findings in each case. (A) Case 1: White villous‐like changes were observed, although their association with ICIs was unclear. (B) Case 2: Erosions and coarse mucosa extending from the duodenum to the jejunum. (C) Case 3: Multiple aphthous ulcers with surrounding erythema were observed. (D, E) Case 4: Multiple erosions and erythema along with circumferential ulcers were observed before vedolizumab (VDZ) administration. (F) Case 4: Mucosal healing and improvement were observed 2 weeks after VDZ administration.

In recent years, cancer immunotherapy, including ICIs such as anti‐CTLA‐4 and anti‐PD‐1/PD‐L1 antibodies, has made remarkable progress. These therapies have demonstrated potent antitumor effects across a variety of malignancies and have been shown to prolong overall survival [1, 2, 3, 4]. The mechanisms underlying the development of imAEs are thought to include T‐cell activation, increased production of inflammatory cytokines, molecular mimicry and cross‐reactivity, autoantibody production mediated by activated B cells, and the direct effects of ICIs [5]. Among imAEs, immune‐related colitis occurs in approximately 30%–40% of patients receiving ICIs of any severity, with Grade 3 or higher colitis accounting for around 10%. Gastrointestinal disorders represent the most common type of Grade 3 or higher imAEs, with colitis particularly frequent in association with CTLA‐4 inhibitors [6]. As ICIs have the potential to induce inflammation in the small intestine, it is important to include evaluation of the small bowel as an integral component of the clinical assessment in patients undergoing ICI therapy [7]. Shimozaki et al. [8] performed SBCE on 23 patients within 60 days of initiating ICI therapy and observed mucosal abnormalities such as erosions and erythema in both the small and large intestines in 14 cases. However, only two of these patients developed Grade 2 or higher immune‐related colitis, and none of the six patients with small intestinal lesions subsequently developed clinically significant colitis. Therefore, the utility of early gastrointestinal surveillance using SBCE for predicting the onset of immune‐related colitis remains controversial. Furthermore, although not reported in SBCE studies, it has been suggested that up to one‐third of immune‐related colitis cases may present with endoscopically normal mucosa. As such, it may be difficult to rule out colitis based solely on endoscopic appearance. However, patients with extensive lesions or deep ulcers have been reported to have a poorer prognosis compared to those with normal mucosa. In addition, the presence of ulcers has been associated with steroid‐refractory disease [9, 10]. In our cases as well, patients with extensive lesions or ulceration required escalation to biologic agents, suggesting that SBCE findings may have potential value in predicting responsiveness to steroid therapy. Delayed endoscopic evaluation, defined as more than 30 days from the onset of enteritis symptoms, has been associated with prolonged corticosteroid use and increased relapse rates. Therefore, endoscopic assessment—including SBCE—should be performed as early as possible after symptom onset. Future studies are needed to further validate the diagnostic and prognostic utility of SBCE in imAE colitis. In particular, the integration of SBCE findings with clinical features, biomarkers, and histopathological data may help stratify disease severity and guide timely therapeutic decisions. Additionally, the development of standardized scoring systems for SBCE findings and the application of artificial intelligence in image interpretation may enhance the clinical utility of this modality in the management of immune‐related gastrointestinal toxicities (Figure 1).

## Consent

The patient signed informed consent for the possible publication of images guaranteeing anonymity.

## Conflicts of Interest

The authors declare no conflicts of interest.

## Data Availability

The authors declare that data supporting the findings of this study are available within the article.
